# Transcriptomic alterations in the heart of non-obese type 2 diabetic Goto-Kakizaki rats

**DOI:** 10.1186/s12933-016-0424-3

**Published:** 2016-08-05

**Authors:** Márta Sárközy, Gergő Szűcs, Veronika Fekete, Márton Pipicz, Katalin Éder, Renáta Gáspár, Andrea Sója, Judit Pipis, Péter Ferdinandy, Csaba Csonka, Tamás Csont

**Affiliations:** 1 Department of Biochemistry, Faculty of Medicine, University of Szeged, Dóm tér 9, Szeged, 6720 Hungary; 2 Department of Physiology, Anatomy and Neuroscience, Faculty of Science and Informatics, University of Szeged, Szeged, Hungary; 3 Department of Genetics, Cell- and Immunobiology, Semmelweis University, Budapest, Hungary; 4 Pharmahungary Group, Szeged, Hungary; 5 Department of Pharmacology and Pharmacotherapy, Semmelweis University, Budapest, Hungary

**Keywords:** Spontaneous diabetes mellitus, Inherited diabetes mellitus, Non-obese type 2 diabetes mellitus, Myocardium, DNA microarray, GO, String, Insulin resistance

## Abstract

**Background:**

There is a spectacular rise in the global prevalence of type 2 diabetes mellitus (T2DM) due to the worldwide obesity epidemic. However, a significant proportion of T2DM patients are non-obese and they also have an increased risk of cardiovascular diseases. As the Goto-Kakizaki (GK) rat is a well-known model of non-obese T2DM, the goal of this study was to investigate the effect of non-obese T2DM on cardiac alterations of the transcriptome in GK rats.

**Methods:**

Fasting blood glucose, serum insulin and cholesterol levels were measured at 7, 11, and 15 weeks of age in male GK and control rats. Oral glucose tolerance test and pancreatic insulin level measurements were performed at 11 weeks of age. At week 15, total RNA was isolated from the myocardium and assayed by rat oligonucleotide microarray for 41,012 genes, and then expression of selected genes was confirmed by qRT-PCR. Gene ontology and protein–protein network analyses were performed to demonstrate potentially characteristic gene alterations and key genes in non-obese T2DM.

**Results:**

Fasting blood glucose, serum insulin and cholesterol levels were significantly increased, glucose tolerance and insulin sensitivity were significantly impaired in GK rats as compared to controls. In hearts of GK rats, 204 genes showed significant up-regulation and 303 genes showed down-regulation as compared to controls according to microarray analysis. Genes with significantly altered expression in the heart due to non-obese T2DM includes functional clusters of metabolism (e.g. *Cyp2e1, Akr1b10*), signal transduction (e.g. Dpp4, *Stat3*), receptors and ion channels (e.g. *Sln*, *Chrng*), membrane and structural proteins (e.g. *Tnni1, Mylk2, Col8a1, Adam33*), cell growth and differentiation (e.g*. Gpc3, Jund*), immune response (e.g. *C3, C4a*), and others (e.g. *Lrp8, Msln, Klkc1, Epn3*). Gene ontology analysis revealed several significantly enriched functional inter-relationships between genes influenced by non-obese T2DM. Protein–protein interaction analysis demonstrated that Stat is a potential key gene influenced by non-obese T2DM.

**Conclusions:**

Non-obese T2DM alters cardiac gene expression profile. The altered genes may be involved in the development of cardiac pathologies and could be potential therapeutic targets in non-obese T2DM.

**Electronic supplementary material:**

The online version of this article (doi:10.1186/s12933-016-0424-3) contains supplementary material, which is available to authorized users.

## Background

Diabetes mellitus is a heterogeneous chronic metabolic disorder characterized by hyperglycemia as a common feature resulting from impaired insulin secretion, insulin resistance, or both [1]. In 2014, the global prevalence of diabetes mellitus (DM) was estimated to be 9 % among adults according to WHO data [2]. The total number of people suffering from DM is projected to almost triple from 190 million to 552 million by 2030 [3, 4]. T2DM accounts for more than 90 % of all diabetes cases and its incidence is continuously rising worldwide [1, 5, 6]. The major cause for this phenomenon is the so-called obesity epidemic due to physical inactivity and increased consumption of energy-rich food [7]. Nevertheless, it is often neglected that around 20 % of T2DM patients are non-obese in Europe and Asia [7–10]. The non-obese T2DM phenotype is characterized by a more pronounced reduction in insulin secretion and less severe insulin resistance as compared to obese T2DM patients [7]. The risk of T2DM in non-obese individuals is considered to be mostly influenced by polygenic inheritance and prenatal environment [7].

It is well known that diabetic patients have an increased risk of developing a number of co-morbidities including cardiovascular diseases (CVD). It has been reported that T2DM patients have a two to fourfold increased risk of CVD in general [7, 11, 12]. Obesity is recognized as an independent risk factor for both T2DM and CVD [7, 13]. Interestingly, it has been reported that non-obese T2DM patients also have a high risk of CVD similarly to that of obese T2DM patients [7, 11, 14]. Indeed, CVD are estimated to be responsible for more than 50 % of deaths among T2DM population [15].

One of the major pathologies of the aforementioned CVD is diabetic cardiomyopathy (DCM) [15, 16]. DCM is defined as left ventricular (LV) diastolic and/or systolic dysfunction with hypertrophy and fibrosis in the absence of preceding hypertension, coronary artery disease and valvular or congenital heart disease [15–17]. Although DCM is a distinct clinical entity, it is also a part of the diabetic atherosclerosis process [18]. DCM might be independent of the coexistence of arterial hypertension, coronary artery disease or other macrovascular complications [18]. DCM is characterized by the development of myocardial damage, reactive hypertrophy and fibrosis, structural and functional changes of the small coronary vessels, and cardiac autonomic neuropathy [18]. These alterations make the diabetic heart more susceptible to ischemia and subsequent remodelling [18–20].

We have previously shown that cardiac gene expression pattern is significantly altered in obese ZDF rats, a model of T2DM and metabolic syndrome [21], and in streptozotocin-induced T1DM rats [22] at the transcript level. The effect of non-obese T2DM on gene expression pattern in various tissue types has been investigated in a few studies. Pancreatic islets [23, 24], liver [25], skeletal muscle [26], adipose tissue [27], hippocampus and prefrontal cortex [28] obtained from the well-known non-obese T2DM model Goto-Kakizaki (GK) rat showed altered gene expression pattern as compared to controls. Surprisingly, whole transcriptomic analysis in the heart of GK rats has not been performed previously. Therefore, in the present study, our aim was to investigate the effect of non-obese T2DM on cardiac alterations of the transcriptome in GK rats.

## Methods

### Ethics approval

This investigation conforms to the National Institutes of Health Guide for the Care and Use of Laboratory Animals (NIH Pub. No. 85–23, Revised 1996) and was approved by the Animal Research Ethics Committee of the University of Szeged.

### Animal model of non-obese T2DM

Male Goto-Kakizaki rats and their age-matched male Wistar controls were obtained from Charles River Laboratories at the age of 6 weeks and were housed at 22 ± 2 °C with a 12:12-h light–dark cycle. The rats received standard rat chow and water ad libitum for 9 weeks after their arrival. The GK rat is a recognized model of inherited type 2 diabetes mellitus [29]. This spontaneously diabetic rat strain was developed by selective breading of Wistar rats with the highest normal blood glucose levels in response to oral glucose tolerance test [30, 31]. GK rats develop a non-obese and mild hyperglycemic phenotype at week 4–5 accompanied by a metabolic state of glucose intolerance and later peripheral insulin resistance [29, 32] which develops to a hyperglycemic insulin-deficient state with aging [29, 32–34]. The metabolic features manifested in this animal model are in many ways similar to the pathogenesis of inherited spontaneous T2DM in humans [29, 32]. However, hyperglycemia and glucose intolerance developed in GK rats are not associated with the development of obesity or hypertension [1]. The adult GK rat of T2DM has been shown to develop cardiovascular complications including left ventricular hypertrophy, fibrosis, as well as diastolic and/or systolic dysfunction [1, 20, 35, 36]. Therefore, the GK rat is an applicable model for investigation of the consequences of non-obese T2DM in the heart.

### Experimental setup

**Fig. 1:**
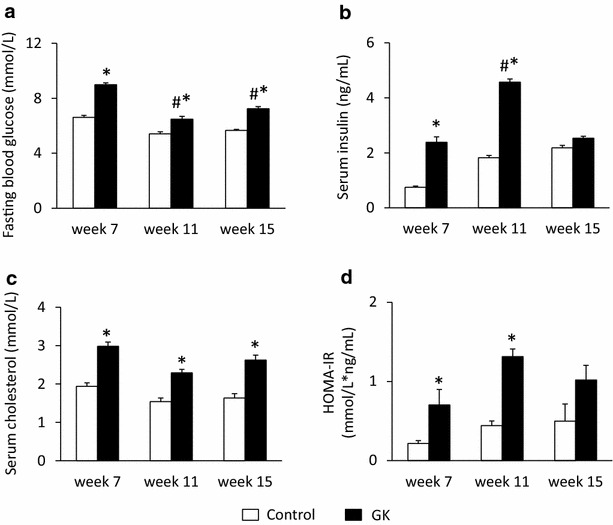
Fasting blood glucose, serum insulin and cholesterol levels. Fasting blood glucose (**a**), serum insulin (**b**) and cholesterol (**c**) levels as well as HOMA-IR index (**d**) at weeks 7, 11 and 15 in both control and GK rats. Values are mean ± SEM, n = 7–9, *p < 0.05 vs. control within the same time point, ^#^p < 0.05 vs. week 7 values

**Fig. 2:**
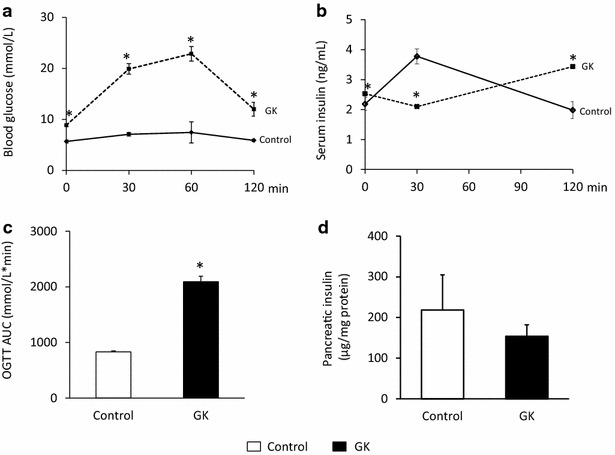
Blood glucose and insulin levels during OGTT and pancreatic insulin content. Blood glucose (**a**) and serum insulin (**b**) levels during OGTT, OGTT AUC (**c**) and pancreatic insulin (**d**) content at week 15 in both control and GK rats. *Solid line* control; *dashed line* GK. Values are mean ± SEM, n = 7–9, *p < 0.05

**Fig. 3:**
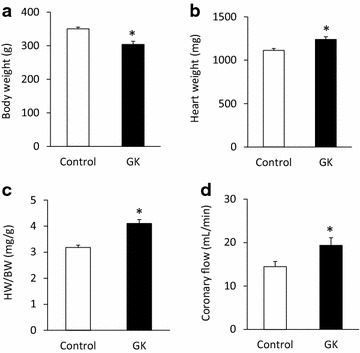
Body weight, heart weight and coronary flow. Body weight (**a**), heart weight (**b**), heart weight to body weight ratio (**c**) and coronary flow (**d**) at week 15 in both control and GK rats. Values are mean ± SEM, n = 7–9, *p < 0.05

Body weight, serum glucose, insulin, cholesterol levels and homeostasis model assessment-estimated insulin resistance (HOMA-IR) were determined at 7, 11 and 15 weeks of age in order to monitor the basic parameters of glucose and lipid metabolism and insulin resistance in GK and control rats (Figs. 1, 2, 3). Oral glucose tolerance test (OGTT) was performed at week 15 to further characterize glucose homeostasis of GK and control rats (Fig. 2). At 15 weeks of age, rats were anaesthetized using pentobarbital sodium (Euthasol, 50 mg/kg, Produlab Pharma b.v., Raamsdonksveer, The Netherlands). Hearts and pancreata were isolated, and then hearts were perfused according to Langendorff as described earlier [21, 37]. After 5 min perfusion ventricular tissue was frozen and stored at −80 °C until gene expression analysis by DNA microarray and qRT-PCR techniques.

### Serum glucose level measurements and OGTT

As described previously, rats were fasted overnight (12 h) prior to serum glucose level measurements and OGTTs (weeks 7, 11 and 15) to verify the development of hyperglycemia and glucose-intolerance as diagnostic criteria of diabetes mellitus [21, 22, 36]. Blood samples were collected from the saphenous vein. Blood glucose levels were measured using AccuCheck blood glucose monitoring systems (Roche Diagnostics Corporation, USA, Indianapolis) [21, 22, 38]. In case of OGTT, after the measurement of baseline glucose concentrations, 1.5 g/kg body weight glucose was administered per os via gavage and blood glucose levels were checked 30, 60 and 120 min later [21, 22, 38]. Area under the curve values for OGTT was also calculated.

### Measurement of serum and pancreatic insulin levels

Serum and pancreatic insulin levels were measured by an enzyme-linked immunosorbent assay (Mercodia, Ultrasensitive Rat Insulin ELISA) as described previously [21, 22, 38]. Blood samples were collected from the saphenous vein at weeks 7, 11 and 15. At week 15, during OGTT blood was collected at 0, 30 and 120 min for serum insulin level measurements. Blood samples were centrifuged (4500 rpm for 10 min at 4 °C) and kept at −20 °C until the assay was performed. At week 15, pancreata were removed, trimmed free of adipose tissue and weighed. Pancreata were homogenized in 6 mL cold acidified-ethanol (0.7 M HCl: ethanol, 1:3 v/v) with an Ultraturrax homogenizer and were kept at 4 °C for 24 h. Then pancreas homogenates were centrifuged (900*g* for 15 min at 4 °C), and the supernatants were stored at 4 °C. The pellet was extracted again with 3 mL acidified ethanol for 24 h at 4 °C. The supernatant obtained after centrifugation was pooled with the previous one and kept at −20 °C until assayed. Insulin ELISA was carried out according to the instructions of the manufacturer from either sera or homogenized pancreatic tissue samples of GK and control rats.

### HOMA-IR index

To estimate insulin resistance in GK or control rats the widely used HOMA-IR index was calculated [21, 39] by multiplying fasting serum insulin (μg/mL) with fasting blood glucose (mmol/L), then dividing by the constant 22.5, i.e. HOMA-IR = (fasting serum insulin concentration × fasting blood glucose concentration)/22.5.

### Measurement of serum cholesterol levels

In order to follow up the development of hypercholesterolemia which is a risk factor of cardiovascular diseases, serum cholesterol levels were measured at weeks 7, 11 and 15 using a test kit (Diagnosticum Zrt., Budapest, Hungary) as described previously [21, 40, 41].

### RNA preparation and DNA microarray analysis

Total RNA was isolated from heart samples with Qiagen miRNeasy Mini Kit according to the manufacturer’s protocol (Qiagen, Hilden, Germany) as described previously [22]. On-column DNase digestion was carried out with the RNase-Free DNase Set (Qiagen GmbH). RNA concentration was measured by NanoDrop 1000 Spectrophotometer (Thermo Fisher Scientific Inc., Waltham, MA, USA) and RNA integrity was determined by an Agilent 2100 Bioanalyzer System (Agilent Technologies Inc., Santa Clara, CA, USA). Samples with an RNA integrity number (RIN) above 8.0 were used for further analysis. RNA was stored at −80 °C until use.

Total RNA (1000 ng) was labelled and amplified using the QuickAmp Labelling Kit according to the instructions of the manufacturer. Labelled RNA was purified and hybridized to Agilent Whole Rat Genome 4 × 44 K array slides, according to the manufacturer’s protocol. After washing, array scanning and feature extraction was performed with default scenario by Agilent DNA Microarray Scanner and Feature Extraction Software 9.5.

### Messenger RNA (mRNA) expression profiling by qRT-PCR

In order to validate gene expression changes obtained by DNA microarray, qRT-PCR was performed with gene-specific primers. Total RNA (1 µg) was reverse transcribed using High-Capacity cDNA Reverse Transcription Kit (Thermo Fisher Scientific, Waltham, MA US). Quantitative RT-PCR was performed using TaqMan Array 96 Well Fast Plate 3 × 32 (Thermo Fisher Scientific, Waltham, MA US) according to the manufacturer’s instructions on a 7900HT Fast Real-Time PCR System. Each well of the TaqMan Array Plate contained 5 μL of Taqman Fast Universal Master Mix (2X) no AmpErase^®^ UNG, 1 μL cDNA (50 ng/μL) and 4 μL distilled water in a final reaction volume of 10 μL per well. Then qPCR was performed with the following protocol: 50 cycles of 95 °C for 15 s and 60 °C for 1 min. The fluorescence intensity was detected after each amplification step. Melting temperature analysis was done after each reaction to check the quality of the products. Primers were designed using the online TaqMan® Assays custom plating service of the manufacturer. Relative expression ratios were calculated as normalized ratios to rat glyceraldehyde-3-phosphate dehydrogenase (GAPDH), hypoxanthine phosphoribosyltransferase (HPRT) and ribosomal protein S18 (RPS18) housekeeping genes. A non-template control sample was used for each primer to check primer-dimer formation. Normalized signal levels for each mRNA were calculated using comparative cycle threshold method (delta–delta Ct method). Fold change refers to 2^−ΔΔCt^ (in the case of up-regulated genes) and −(1/2^−ΔΔCt^) (in the case of down-regulated genes).

### Gene ontology (GO) analysis

By using DNA microarrays for transcriptional profiling a large number of genes can be analyzed simultaneously [21, 42], however, the resulting data do not give direct information about possible biological interaction of the differentially expressed genes. GO analysis is a suitable method for integration genes with pathways and biological interaction networks to detect coordinated changes in functionally related genes [21, 42]. GO analysis was performed using GO pathway analysis using the open access software DAVID bioinformatics system and database (Database for Annotation, Visualization and Integrated Discovery, http://www.david.abcc.ncifcrf.gov website) [21, 42]. The differentially expressed genes were submitted to DAVID bioinformatics system and database to reveal significantly enriched biological functions/pathways [21, 42].

### Protein–protein interaction analysis

Gene expression networks are of great biological interest since co-expressed genes could be (1) controlled by the same transcriptional regulatory program, (2) functionally related, or (3) members of the same pathway or protein complex [43]. In order to further characterize the connections of significantly altered genes obtained by DNA microarray, protein–protein network analysis was performed by STRING10 based on two types of evidence: experimental (protein–protein interaction databases) and text-mining (abstracts of scientific literature). STRING (http://www.string-db.org/), is an online accessible database of known and predicted protein–protein interactions. Protein–protein interactions from STRING 10 were collected for the construction of differential protein interaction network among the genes whose expressions were significantly different in hearts of GK rats. The differently expressed genes were mapped to the String database and then known and predicted associations were scored and integrated. Combined-score > 0.4 was the threshold. Differently expressed genes were visualized after KMEANS clustering. Finally, interaction network was constructed by integrating these relationships.

### Statistical analysis

Statistical analysis was performed by using Sigmaplot 12.0 for Windows (Systat Software Inc). All values are presented as mean ± SEM. Repeated measures Two-Way ANOVA was used to determine the effect of T2DM and the age on FBG, serum insulin and cholesterol levels as well as glucose levels during OGTT. After ANOVA, all pairwise multiple comparison procedures with Holm-Šídák post hoc tests were used as multiple range tests. Two sample t test was used to determine the effect of T2DM on OGTT AUC, pancreatic insulin concentration, body weight, heart weight, heart weight/body weight ratio and coronary flow. P < 0.05 was accepted as a statistically significant difference. In the microarray experiments, biological and technical replica tests were carried out to gain raw data for statistical analysis. Altogether 4 individual parallel gene activity comparisons were done between the two groups. Both in the microarray and qRT-PCR experiments, a two-sample t test was used and the p value was determined to find significant gene expression changes. In the microarray experiments, a corrected p value was determined for each gene to control the false discovery rate using the Benjamini and Hochberg multiple testing correction protocol. Gene expression ratios with p value of <0.05 and log_2_ ratio of < −1.00 or log_2_ ratio of >1.00 (~2.0-fold were considered as repression or overexpression respectively in gene activity.

## Results

### Metabolic characterization of non-obese T2DM

In order to verify the development of T2DM in male GK rats, concentrations of several serum metabolites were measured at weeks 7, 11 and 15 (Fig. 1). GK rats showed a significantly elevated FBG level at all time points as compared to controls (Fig. 1a). Interestingly, blood glucose level in GK rats were significantly lower at week 11 as compared to week 7 or week 15 blood glucose values (Fig. 1a). Parallel with hyperglycemia, serum insulin levels were significantly increased in GK rats compared to Wistar controls at week 7 and more profoundly at week 11 showing the presence of hyperinsulinemia in GK animals (Fig. 1b). However, there was no significant difference between serum insulin levels measured in GK and control rats at week 15, since serum insulin concentration in GK rats significantly decreased by week 15 as compared to insulin level measured at week 11 indicating beta-cell damage in GK rats (Fig. 1b). HOMA-IR was significantly increased at weeks 7 and 11 in GK rats when compared to controls showing insulin resistance in GK animals (Fig. 1d). In contrast, increase of HOMA-IR did not reach the level of statistical significance at week 15 in GK animals (Fig. 1d). Serum cholesterol levels were significantly higher in GK rats as compared to control ones throughout the study duration representing hypercholesterolemia (Fig. 1c). OGTT was performed at week 15 in order to verify the development of impaired glucose tolerance in GK rats. Glucose levels during OGTT were markedly increased in GK rats in every time point of blood glucose measurements (Fig. 2a). Area under the curve (AUC) of blood glucose levels during OGTT was significantly increased in GK rats representing impaired glucose tolerance (Fig. 2c). In addition, serum insulin level in GK rats during OGTT was significantly lower 30 min after glucose loading and became markedly increased 120 min after glucose administration indicating impaired insulin secretion (Fig. 2b). Interestingly, pancreatic insulin levels were 25 % lower in GK rats compared to control ones, however, the values were not significantly different between the two groups (Fig. 2d).

### Body weight, heart weight and coronary flow

Body weight was significantly decreased at week 15 in GK rats proving a non-obese phenotype of T2DM in GK rats (Fig. 3a). Heart weight and heart weight to body weight ratio were significantly increased at week 15 in GK rats suggesting the development of cardiac hypertrophy in response to chronic T2DM (Fig. 3b, c). Interestingly, coronary flow was significantly increased at week 15 in GK rats as compared to control hearts (Fig. 3d).

### Gene expression profile and qRT-PCR

Among the 41,012 genes surveyed (Additional file 1: Table S1), 507 genes whose expression was > ~2.0-fold up- or down-regulated in hearts of GK rats relative to levels of control rats showed significant change in expression. According to our results, 204 genes showed up-regulation (Additional file 2: Table S2) and 303 genes showed down-regulation in hearts of GK rats (Additional file 2: Table S3). Moreover, 138 genes showed more than threefold change of expression in hearts of GK rats as compared to the levels of control rats. Among these 138 genes, 50 genes were significantly up-regulated (Table 1) and 88 genes were significantly down-regulated (Table 2) in GK rat hearts. The expression change of selected 28 genes was validated by qRT-PCR (Table 3), 19 of these 28 genes have been confirmed by qRT-PCR (Table 3).

**Table 1:** Up-regulated genes (>3.0-fold up-regulation)

Gene function	Description (gene symbol)	Acc. no.	AVE log2	SD (LOG)	P value (Corr)	Fold change	SD
Metabolism	Abhydrolase domain containing 17C (Abhd17c)	NM_001100736	2.79	0.39	0.00	6.96	1.72
	Alcohol dehydrogenase 1 (class I) (Adh1)	NM_019286	2.43	0.99	0.00	5.38	2.78
	Retinol saturase (all trans retinol 13,14 reductase) (Retsat)	NM_145084	2.17	1.14	0.01	4.50	3.61
	Diacylglycerol kinase, beta (Dgkb)	NM_019304	2.07	0.34	0.00	4.21	0.82
	Kallikrein 1-related peptidase C3 (Klk1c3)	NM_001271315	1.92	0.45	0.00	3.79	1.07
	Aldo–keto reductase family 1, member B10 (aldose reductase) (Akr1b10)	NM_001013084	1.91	0.44	0.00	3.76	1.05
	Adenosine monophosphate deaminase 1 (Ampd1)	NM_138876	1.61	0.71	0.00	3.06	1.56
Signal transduction, regulation of transcription	Phospholipase A2, group VII (platelet-activating factor acetylhydrolase, plasma) (Pla2g7)	NM_001009353	2.23	0.29	0.00	4.68	0.88
	Protein tyrosine phosphatase, non-receptor type 13 (Ptpn13)	NM_001100789	2.13	1.06	0.00	4.38	2.18
	Ephrin A2 (Efna2)	NM_001168670	1.77	0.31	0.00	3.41	0.60
	Crystallin, mu (Crym)	NM_053955	1.76	0.39	0.00	3.38	0.78
	Cd47 molecule (Cd47)	NM_019195	1.74	0.26	0.00	3.34	0.51
	Calsyntenin 2 (Clstn2)	NM_134377	1.63	0.65	0.00	3.09	1.41
	Ring finger protein 187 (Rnf187)	NM_001164264	1.61	0.34	0.00	3.06	0.63
Receptors, ion channels	Potassium voltage-gated channel, Isk-related family, member 1 (Kcne1)	NM_012973	2.72	0.82	0.00	6.57	4.07
	ATPase, H+ transporting, lysosomal V1 subunit G2 (Atp6v1g2)	NM_212490	2.53	0.60	0.00	5.77	2.02
	Cholinergic receptor, nicotinic, alpha 7 (neuronal) (Chrna7)	S53987	1.79	0.31	0.00	3.45	0.63
	ATPase, Ca++ transporting, plasma membrane 2 (Atp2b2)	NM_012508	1.71	0.50	0.00	3.27	0.91
Membrane proteins	Peroxisomal membrane protein 4 (Pxmp4)	NM_172223	2.52	0.21	0.00	5.73	0.74
Cell growth and differentiation	Transcription elongation factor A (SII)-like 7 (Tceal7)	NM_001109401	1.86	0.84	0.00	3.63	1.88
	Jun D proto-oncogene (Jund)	NM_138875	1.71	0.29	0.00	3.28	0.60
Immune response	RT1 class Ib, locus T18 (RT1-T18)	NM_001002821	2.31	0.77	0.00	4.95	2.21
	2′-5′ oligoadenylate synthetase 1A (Oas1a)	NM_138913	1.98	0.86	0.00	3.94	2.50
Transport	ATPase, Ca++ transporting, plasma membrane 2 (Atp2b2)	NM_012508	1.78	0.41	0.00	3.44	0.77
	Solute carrier family 16 (monocarboxylate transporter), member 7 (Slc16a7)	NM_017302	1.64	0.38	0.00	3.11	0.69
Others	Guanylate binding protein 1, interferon-inducible (gbp1)	XM_006224278	4.80	0.49	0.00	27.84	8.96
	Uncharacterized LOC102549588	XR_362031	4.69	0.54	0.00	25.85	8.31
	Twin-arginine translocation pathway signal		4.52	0.58	0.00	23.01	9.81
	TL0ADA29YA08 mRNA sequence	FQ222105	3.27	0.42	0.00	9.64	2.34
	Similar to ribosomal protein L6 (LOC366145)	XM_345411	2.94	0.59	0.00	7.70	3.03
	Mitochondrial ribosomal protein S10 (Mrps10)	NM_001008859	2.86	1.24	0.00	7.28	4.07
	Cd300 molecule-like family member G (Cd300lg)	XM_003750936	2.61	0.68	0.00	6.10	2.99
	MRNA decapping enzyme		2.32	0.35	0.00	4.99	1.11
	Transmembrane emp24 domain-containing protein 5 precursor		2.14	0.28	0.00	4.41	0.72
	Macrophage activation 2 like (Mpa2 l)	XM_006221662	2.10	1.05	0.00	4.30	3.00
	Similar to interferon-inducible GTPase (RGD1309362)	NM_001024884	2.02	0.84	0.00	4.06	2.65
	Myc-like oncogene, s-myc protein (Mycs)	NM_021837	2.00	0.80	0.00	4.00	1.64
	Uncharacterized LOC102556738	XR_340771	1.96	0.44	0.00	3.89	1.10
	Nuclear casein kinase and cyclin-dependent kinase substrate 1 (Nucks1)	XM_006249797	1.96	0.34	0.00	3.89	0.74
	Transducin-like enhancer of split 6 (E(sp1) homolog, Drosophila) (Tle6)	XM_006241027	1.95	0.43	0.00	3.87	1.10
	Transmembrane protein 132C (Tmem132c)	XM_002724836	1.91	0.45	0.00	3.75	1.09
	CWC25 spliceosome-associated protein homolog (*S. cerevisiae*) (Cwc25)	NM_001108295	1.82	0.76	0.00	3.52	1.65
	Contactin associated protein-like 2 (Cntnap2)	XM_006236412	1.79	0.39	0.00	3.47	0.84
	Anaphase promoting complex subunit 10 (Anapc10)	XM_006255406	1.77	1.29	0.03	3.41	4.41
	Deoxycytidine triphosphate deaminase		1.73	0.35	0.00	3.32	0.74
	Hypothetical protein LOC500028	NM_001047954	1.65	0.38	0.00	3.13	0.66
	RNA binding motif protein 44 (Rbm44)	XM_001066845	1.64	0.46	0.00	3.12	0.87
	Trichorhinophalangeal syndrome I (Trps1)	XM_006241626	1.62	0.39	0.00	3.08	0.69
	Excision repair cross-complementing rodent repair deficiency, complementation group 8 (Ercc8)	NM_001107650	1.60	0.27	0.00	3.03	0.49
	Uncharacterized LOC100912569	XR_348427	1.59	0.82	0.01	3.02	1.80

**Table 2:** Down-regulated genes (>3.0-fold down-regulation)

Gene function	Description (gene symbol)	Acc. no.	AVE log2	SD (LOG)	P value (Corr)	Fold change	SD
Metabolism	Transglutaminase 1 (Tgm1)	NM_031659	−1.98	0.55	0.0002	−3.93	1.87
	CARBONIC anhydrase 6 (Car6)	NM_001134841	−2.03	0.76	0.0002	−4.08	2.61
	Phenazine biosynthesis-like protein domain containing 1 (Pbld1)	NM_138530	−2.14	0.68	0.0001	−4.41	2.60
	Coproporphyrinogen oxidase (Cpox)	NM_001037095	−2.32	0.45	0.0001	−4.98	2.06
	Prolyl 4-hydroxylase, alpha polypeptide III (P4ha3)	XM_006229769	−2.36	0.38	0.0001	−5.13	1.68
Signal transduction, regulation of transcription	cAMP responsive element binding protein 5 (Creb5)	XM_006236505	−1.62	0.52	0.0001	−3.07	1.26
	Dual-specificity tyrosine-(Y)-phosphorylation regulated kinase 3 (Dyrk3)	NM_001024767	−1.74	0.65	0.0006	−3.35	1.95
	Hypermethylated in cancer 1 (Hic1)	NM_001107021	−1.83	0.70	0.0019	−3.55	2.10
	Rho guanine nucleotide exchange factor (GEF) 5 (Arhgef5)	XM_006224892	−1.96	0.47	0.0001	−3.89	1.45
	Thyroid hormone responsive (Thrsp)	NM_012703	−2.28	0.98	0.0020	−4.86	5.18
	Cellular retinoic acid binding protein 2 (Crabp2)	NM_017244	−2.32	0.50	0.0001	−4.98	2.42
	A kinase (PRKA) anchor protein 3 (Akap3)	NM_001005557	−2.68	0.35	0.0000	−6.39	1.90
	Uroplakin 1B (Upk1b)	NM_001024253	−3.69	0.69	0.0001	−12.89	7.94
Receptors, ion channels	Interleukin 22 receptor, alpha 2 (Il22ra2)	NM_001003404	−1.62	0.93	0.0064	−3.07	2.78
	Neurotrophic tyrosine kinase, receptor, type 3 (Ntrk3), transcript variant 3	NM_019248	−1.64	0.45	0.0005	−3.12	1.18
	Adrenoceptor alpha 1D (Adra1d)	NM_024483	−1.69	0.37	0.0007	−3.23	1.09
	Lysophosphatidic acid receptor 1 (Lpar1)	NM_053936	−1.83	0.51	0.0000	−3.55	1.50
	Prostaglandin F receptor (Ptgfr)	NM_013115	−1.87	0.44	0.0000	−3.65	1.40
	Solute carrier family 4 (anion exchanger), member 1 (Slc4a1)	NM_012651	−2.39	0.22	0.0000	−5.23	0.94
	Sarcolipin (Sln)	NM_001013247	−3.00	1.41	0.0028	−8.03	15.41
	ATPase, H + transporting, lysosomal accessory protein 1-like (Atp6ap1 l)	NM_001191843	−3.59	0.31	0.0000	−12.06	3.25
Membrane proteins	Sarcolipin (Sln)	CK841541	−2.51	1.08	0.0019	−5.68	7.12
Structural protein, cell adhesion	Collagen, type V, alpha 3 (Col5a3)	NM_021760	−1.78	0.59	0.0001	−3.43	1.46
	Contactin associated protein 1 (Cntnap1)	NM_032061	−2.03	0.34	0.0000	−4.09	1.11
	Myosin binding protein H-like (Mybphl)	NM_001014042	−2.82	1.17	0.0011	−7.08	8.55
	Myosin, light chain 7, regulatory (Myl7)	NM_001106017	−3.37	1.66	0.0030	−10.34	20.29
	Mesothelin (Msln)	NM_031658	−4.46	1.44	0.0010	−22.03	31.18
Cell growth and differentiation	Ret proto-oncogene (Ret), transcript variant 1	NM_012643	−1.64	0.44	0.0000	−3.11	1.15
	Endothelial cell-specific molecule 1 (Esm1)	NM_022604	−2.28	0.39	0.0000	−4.85	1.57
	Neuronatin (Nnat), transcript variant 1	NM_053601	−2.34	1.34	0.0119	−5.06	10.47
	Endothelial cell-specific molecule 1 (Esm1)	NM_022604	−2.36	0.40	0.0000	−5.13	1.64
	Epiphycan (Epyc)	NM_001108088	−2.36	1.37	0.0083	−5.14	7.64
	Cyclin G1 (Ccng1)	NM_012923	−3.36	0.21	0.0000	−10.29	1.82
	Tripartite motif-containing 16 (Trim16)	NM_001135033	−6.33	0.41	0.0000	−80.63	28.07
Immune response	Regenerating islet-derived 3 beta (Reg3b)	NM_053289	−1.81	1.34	0.0300	−3.52	5.83
	Inducible T-cell co-stimulator ligand (Icoslg)	XM_006223832	−1.92	0.37	0.0000	−3.79	1.16
	Complement component 4A (Rodgers blood group) (C4a)	NM_031504	−2.25	0.75	0.0002	−4.77	3.07
	Chemokine (C-X-C motif) ligand 13 (Cxcl13)	NM_001017496	−2.48	0.78	0.0003	−5.57	4.38
	Similar to immunoglobulin superfamily, member 7 (RGD1559482),	NM_001168285	−2.54	0.43	0.0000	−5.83	2.19
	CD1d1 molecule (Cd1d1)	NM_017079	−2.64	0.53	0.0000	−6.21	2.67
	Chymase 1, mast cell (Cma1)	NM_013092	−3.95	0.45	0.0000	−15.40	6.26
Transport	Retinol binding protein 4, plasma (Rbp4)	NM_013162	−2.09	1.20	0.0131	−4.27	7.22
Hormones	Inhibin alpha (Inha)	NM_012590	−1.79	0.56	0.0020	−3.45	1.80
Others	Protein Arhgef5 (Source:UniProtKB/TrEMBL;Acc:E9PT59)	XM_006224892	−1.62	0.54	0.0002	−3.07	1.33
	Hypothetical protein LOC100302372 (LOC100302372)	NM_001162897	−1.63	0.57	0.0004	−3.10	1.69
	(F344/Crj)rearranged mRNA for T-cell receptor gamma chain (1483 bp)	Z27087	−1.65	0.71	0.0045	−3.13	1.94
	Similar to RIKEN cDNA 1700001E04 (LOC367428), mRNA (XM_346135)	XM_346135	−1.65	0.25	0.0000	−3.14	0.68
	BTB (POZ) domain containing 9 (Btbd9)	XM_006256185	−1.66	0.30	0.0000	−3.15	0.73
	Q99NG8_RAT (Q99NG8) T:G mismatch thymine glycosylase		−1.67	0.44	0.0001	−3.17	1.24
	Hypothetical protein LOC689316 (LOC689316)	XR_086061	−1.68	0.39	0.0000	−3.21	1.02
	Uroplakin 3B-like (Upk3bl)	NM_001109020	−1.69	0.37	0.0013	−3.22	1.04
	NEUU_MOUSE (Q9QXK8) Neuromedin U-23 precursor (NmU-23)		−1.71	0.43	0.0000	−3.28	1.10
	Ripply transcriptional repressor 2 (Ripply2)	XM_001064780	−1.73	0.44	0.0000	−3.31	1.22
	Uncharacterized LOC100912446 (LOC100912446)	FQ221838	−1.73	0.64	0.0005	−3.33	1.76
	Similar to TP53-regulating kinase (p53-related protein kinase) (Nori-2) (LOC685619)	XM_002729250	−1.75	0.32	0.0000	−3.37	0.84
	Erythrocyte membrane protein band 4.1-like 3 (Epb41l3)	NM_053927	−1.77	0.90	0.0057	−3.41	2.69
	Uncharacterized LOC102556259 (LOC102556259)	XR_355327	−1.77	0.38	0.0001	−3.42	1.22
	EF-hand domain family, member D1 (Efhd1)	NM_001109310	−1.81	0.46	0.0000	−3.50	1.45
	Zinc finger and BTB domain containing 20 (Zbtb20)	XM_006248302	−1.87	0.48	0.0000	−3.67	1.69
	Suppressor of glucose, autophagy associated 1 (Soga1)	XM_001067659	−1.89	0.66	0.0001	−3.72	2.23
	Protein RGD1562667	XM_221091	−1.91	0.45	0.0000	−3.75	1.49
	Uncharacterized protein (Source:UniProtKB/TrEMBL;Acc:F1LSJ2) (ENSRNOT00000035259)	XM_001061015	−1.93	0.61	0.0001	−3.80	2.07
	Uncharacterized LOC102546664 (LOC102546664)	XR_342060	−1.95	0.18	0.0000	−3.87	0.58
	Protein Rsf1 (Source:UniProtKB/TrEMBL;Acc:D3ZGQ8)	XM_218939	−1.95	1.05	0.0109	−3.87	2.89
	Nucleosome assembly protein 1-like 5 (Nap1l5)	NM_001044293	−1.97	1.04	0.0039	−3.92	3.86
	Methyltransferase like 2B (Mettl2b)	NM_001108839	−2.07	0.40	0.0000	−4.20	1.36
	Similar to immunoreceptor Ly49si3 (LOC690097)	XM_003753951	−2.19	0.50	0.0001	−4.55	2.20
	Neuronal PAS domain protein 2 (Npas2)	NM_001108214	−2.23	0.47	0.0000	−4.69	1.73
	FM089532 etnofat cDNA clone etnofatP0014D18 5′, mRNA sequence	FM089532	−2.40	0.36	0.0001	−5.28	1.54
	Chordin-like 1 (Chrdl1)	NM_199502	−2.46	0.27	0.0000	−5.51	1.30
	Uncharacterized LOC102557390 (LOC102557390)	XR_348511	−2.54	0.48	0.0000	−5.82	2.30
	Q7TQ12_RAT (Q7TQ12) Aa1114		−2.62	0.32	0.0000	−6.14	1.69
	TL0ACA45YL24 mRNA sequence	FQ215947	−2.65	0.48	0.0000	−6.27	2.81
	Aryl hydrocarbon receptor nuclear translocator-like (Arntl)	NM_024362	−2.75	0.54	0.0000	−6.75	2.85
	Similar to RIKEN cDNA 1500015O10 (RGD1305645)	NM_001271051	−2.84	0.88	0.0003	−7.17	7.06
	Uncharacterized LOC100911508 (LOC100911508)	XR_145872	−2.86	0.30	0.0000	−7.25	1.86
	TL0ACA40YB18 mRNA sequence.	FQ216879	−2.99	1.21	0.0012	−7.94	9.21
	Family with sequence similarity 216, member B (Fam216b)	XM_003751515	−3.04	0.89	0.0002	−8.25	6.41
	SARCO_MOUSE (Q9CQD6) Sarcolipin, complete (TC628765)	AW918768	−3.06	1.35	0.0020	−8.33	14.57
	Endogenous retrovirus mRNA	AY212271	−3.51	1.47	0.0015	−11.38	17.10
	Uncharacterized LOC102552170 (LOC102552170)	XM_006224493	−4.01	0.40	0.0000	−16.09	5.74
	O89816_9GAMR (O89816) Envelope glycoprotein		−4.04	0.99	0.0001	−16.49	15.56
	Rat PRRHIS8 mRNA for ribosomal protein S8. (X56846)	X56846	−4.14	0.73	0.0000	−17.63	12.05
	Elongator protein 3/MiaB/NifB		−4.21	0.37	0.0000	−18.51	5.56
	Endogenous retrovirus mRNA	AY212271	−4.21	1.48	0.0006	−18.52	28.67
	Similar to 60S ribosomal protein L19 (LOC316856)	XM_229366	−4.46	0.84	0.0000	−22.05	16.63
	Uncharacterized LOC102554872 (LOC102554872)	XR_348916	−4.86	0.35	0.0000	−29.02	8.07
	WDNM1 homolog (LOC360228)	NM_001003706	−5.14	1.80	0.0013	−35.24	76.86

**Table 3:** Confirmation by qRT-PCR

Gene symbol	Gene name	Acc. nr.	DNA microarray		qRT-PCR		Confirmed
			AVE (log2)	Fold change	Fold change	SEM	
Adipoq	Adiponectin, C1Q and collagen domain containing	Rn00595250_m1	−4.99	−31.78	−23.64	0.02	Yes
Retn	Resistin	Rn00595224_m1	−4.74	−26.72	31.08	14.46	No
Atp1b4	ATPase, (Na+)/K+ transporting, beta 4 polypeptide	Rn00584523_m1	−4.21	−18.51	−4.74	0.05	Yes
Car3	Carbonic anhydrase 3	Rn01461970_m1	−3.73	−13.27	−7.88	0.03	Yes
Cma1	Chymase 1, mast cell	Rn00565319_m1	−3.11	−8.63	−5.12	0.02	Yes
Arntl	Aryl hydrocarbon receptor nuclear translocator-like	Rn00577590_m1	−2.75	−6.74	−2.95	0.03	Yes
Tgm1	Transglutaminase 1, K polypeptide	Rn00581408_m1	−1.97	−3.92	−4.88	0.03	Yes
Nnat	Neuronatin (Nnat), transcript variant 1	Rn00440480_m1	−1.87	−3.66	−4.2	0.02	Yes
Ddah1	Dimethylarginine dimethylaminohydrolase 1	Rn00574200_m1	−1.69	−3.23	−3.36	0.02	Yes
Ntrk3	Neurotrophic tyrosine kinase, receptor, type 3	Rn00570389_m1	−1.69	−3.23	−2.33	0.06	Yes
Stat3	Signal transducer and activator of transcription 3	Rn00562562_m1	−1.49	−2.81	1.36	0.08	No
Dpp4	Dipeptidylpeptidase 4	Rn00562910_m1	−1.35	−2.55	−2.11	0.03	Yes
Ephx2	Epoxide hydrolase 2, cytoplasmic	Rn00576023_m1	−1.33	−2.51	−4.23	0.02	Yes
Gpc3	Glypican 3	Rn00516722_m1	−1.29	−2.45	−2.54	0.04	Yes
Fgf18	Fibroblast growth factor 18	Rn00433286_m1	−1.17	−2.25	−2.36	0.03	Yes
Tfpi	Tissue factor pathway inhibitor (lipoprotein-associated coagulation inhibitor)	Rn00567935_m1	−1.10	−2.14	1.12	0.06	No
Gckr	Glucokinase (hexokinase 4) regulator	Rn00565467_m1	−0.98	−1.97	−2.09	0.05	N/A
Cdkn1a	Cyclin-dependent kinase inhibitor 1A	Rn00589996_m1	−0.93	−1.91	−1.34	0.04	N/A
Bat5	HLA-B associated transcript 5	Rn01525709_g1	0.96	1.95	−1.3	0.06	N/A
Sele	Selectin E	Rn00594072_m1	1.02	2.03	2.35	0.14	Yes
Dbp	D site of albumin promoter (albumin D-box) binding protein	Rn00497539_m1	1.06	2.08	2.39	0.17	Yes
Abcg1	ATP-binding cassette, subfamily G (WHITE), member 1	Rn00585262_m1	1.08	2.11	1.99	0.14	No
Cyr61	Cysteine-rich, angiogenic inducer, 61	Rn00580055_m1	1.14	2.20	1.58	0.12	No
Ephx1	Epoxide hydrolase 1, microsomal	Rn00563349_m1	1.21	2.31	2.24	0.18	Yes
Prkce	Protein kinase C, epsilon	Rn01769089_m1	1.28	2.43	1.09	0.08	No
Nurp1	Nuclear protein, transcriptional regulator, 1	Rn00586046_m1	1.34	2.53	3.29	0.28	Yes
Slc16a7	Solute carrier family 16, member 7 (monocarboxylic acid transporter 2)	Rn00568872_m1	1.77	3.41	3.21	0.19	Yes
Atp2b2	ATPase, C++ transporting, plasma membrane 2	Rn01425460_m1	1.88	3.68	3.78	0.21	Yes

### Gene ontology analysis

In order to further determine the biological significance and functional classification of differentially expressed genes due to non-obese T2DM, GO analysis was performed (Table 4) [21]. GO analysis is suitable for identifying significantly enriched GO terms related to multiple genes and for discovering enriched functionally related gene groups. A single gene can belong to different categories. Out of the 507 genes significantly altered by non-obese T2DM in our present study, 277 genes with known function were submitted to GO analysis and 115 were clustered into different categories. The rest of the 507 genes were either unknown expressed sequence tags or unrecognized by the GO analysis database (Table 4). The 115 analyzed genes were classified into five main categories such as (1) biological regulation, (2) metabolic process, (3) immune system process, (4) biological adhesion, and (5) rhythmic process (Table 4).

**Table 4:** Gene ontology analysis

Category	Term	Count	%	P value	Gene symbol
**GOTERM_BP_1**	**GO:0065007** **~** **biological regulation**	**48**	**41.74**	**0.01**	**FGF18, PDIA2, GPBP1, C3, CRABP2, TRIM16, ZEB2, LPAR1, ESM1, SGMS1, CCNG1, CD1D1, ALDH1A1, ATP2B2, CD47, NPAS2, DGKB, ZFP90, CXCR6, JUND, PER2, TEF, CHRNA7, CNTNAP1, TRAF6, QSOX1,DDAH1, DPP4, DPT, CYR61, RET, NCF1, NUCKS1, ARHGEF5, PRKAB2, LOC501307, ARNTL, PTGFR, PRKCE, TMEM189, STAT3, FMN1, TRPS1, KLRE1, GNB3, EIF2AK2, ICOSLG, BARD1**
**GOTERM_BP_2**	**GO:0006950** **~** **response to stress**	**16**	**13.91**	**0.04**	**NCF1, PDIA2, C3, SGMS1, CD1D1, STAT3, ALDH1A1, ERCC8, SDC1, PLOD2, CHRNA7, TRAF6, EIF2AK2, DDAH1, DPP4, BARD1**
**GOTERM_BP_2**	**GO:0050789** **~** **regulation of biological process**	**45**	**39.13**	**0.02**	**FGF18, PDIA2, GPBP1, C3, TRIM16, ZEB2, LPAR1, ESM1, SGMS1, CD1D1, ALDH1A1, ATP2B2, CD47, NPAS2, DGKB, ZFP90, CXCR6, JUND, PER2, TEF, CHRNA7, CNTNAP1, TRAF6, QSOX1, DDAH1, DPP4, DPT, CYR61, RET, NCF1, NUCKS1, ARHGEF5, PRKAB2, ARNTL, PTGFR, PRKCE, TMEM189, STAT3, FMN1, TRPS1, KLRE1, GNB3, EIF2AK2, ICOSLG, BARD1**
**GOTERM_BP_2**	**GO:0048518** **~** **positive regulation of biological process**	**24**	**20.87**	**0.00**	**FGF18, C3, GPBP1, ZEB2, TRIM16, ARNTL, LPAR1, CD1D1, STAT3, FMN1, ALDH1A1, ATP2B2, CD47, NPAS2, JUND, KLRE1, TEF, CHRNA7, EIF2AK2, TRAF6, DDAH1, ICOSLG, CYR61, BARD1**
**GOTERM_BP_2**	**GO:0048646** **~** **anatomical structure formation involved in morphogenesis**	**9**	**7.83**	**0.00**	**ALDH1A1, ATP2B2, FGF18, RET, RIPPLY2, ZEB2, TRAF6, DDAH1, CYR61**
**GOTERM_BP_2**	**GO:0009653** **~** **anatomical structure morphogenesis**	**14**	**12.17**	**0.03**	**FMN1, ALDH1A1, ATP2B2, FGF18, SDC1, RET, EFNA2, CRABP2, RIPPLY2, ZEB2, TRAF6, DDAH1, STAT3, CYR61**
**GOTERM_BP_1**	**GO:0008152** **~** **metabolic process**	**48**	**41.74**	**0.03**	**CYP2J4, OCLN, C3, CRABP2, TRIM50, TRIM16, LPAR1, SGMS1, ALDH1A1, ATP2B2, ERCC8, MCM8, ST6GALNAC3, PLOD2, ZFP90, CPOX, P4HA3, JUND, PER2, TEF, CHRNA7, TRAF6, QSOX1, DDAH1, DPP4, RET, NCF1, PRKAB2, LOC501307, ARNTL, CRYZ, TMEM189, LPCAT2, PRKCE, WEE1, STAT3, RPS7, OXSM, FMN1, TAF13, SLC16A7, AKR1B10, PLA2G7, CMA1, EIF2AK2, CAR6, PRPS2, BARD1**
**GOTERM_BP_2**	**GO:0044237** **~** **cellular metabolic process**	**39**	**33.91**	**0.09**	**CYP2J4, OCLN, CRABP2, TRIM50, TRIM16, LPAR1, SGMS1, ALDH1A1, ATP2B2, ERCC8, MCM8, ST6GALNAC3, ZFP90, CPOX, JUND, PER2, TEF, CHRNA7, TRAF6, QSOX1, DDAH1, RET, NCF1, PRKAB2, LOC501307, ARNTL, TMEM189, PRKCE, LPCAT2, WEE1, STAT3, RPS7, OXSM, TAF13, SLC16A7, EIF2AK2, CAR6, PRPS2, BARD1**
*GOTERM_BP_3*	*GO:0034641* ~ *cellular nitrogen compound metabolic process*	*19*	*16.52*	*0.09*	*OCLN, LOC501307, ARNTL, LPCAT2, STAT3, RPS7, ERCC8, ATP2B2, MCM8, TAF13, ZFP90, CPOX, JUND, TEF, PER2, CHRNA7, DDAH1, PRPS2, BARD1*
GOTERM_BP_4	GO:0006575 ~ cellular amino acid derivative metabolic process	4	3.48	0.10	ATP2B2, OCLN, CHRNA7, LPCAT2
*GOTERM_BP_3*	*GO:0010033* ~ *response to organic substance*	*11*	*9.57*	*0.09*	*ALDH1A1, CYP2J4, SDC1, JUND, TRIM16, CHRNA7, GNB3, EIF2AK2, DDAH1, STAT3, CYR61*
GOTERM_BP_4	GO:0014070 ~ response to organic cyclic substance	5	4.35	0.05	ALDH1A1, CYP2J4, JUND, CHRNA7, STAT3
*GOTERM_BP_3*	*GO:0006629* ~ *lipid metabolic process*	*9*	*7.83*	*0.08*	*ALDH1A1, CYP2J4, NCF1, CRABP2, PRKAB2, PLA2G7, SGMS1, LPCAT2, OXSM*
GOTERM_BP_4	GO:0006720 ~ isoprenoid metabolic process	3	2.61	0.06	ALDH1A1, CYP2J4, CRABP2
GOTERM_BP_5	GO:0006720 ~ isoprenoid metabolic process	3	2.61	0.05	ALDH1A1, CYP2J4, CRABP2
GOTERM_BP_5	GO:0001523 ~ retinoid metabolic process	3	2.61	0.02	ALDH1A1, CYP2J4, CRABP2
GOTERM_BP_4	GO:0042573 ~ retinoic acid metabolic process	2	1.74	0.10	ALDH1A1, CRABP2
*GOTERM_BP_3*	*GO:0044255* ~ *cellular lipid metabolic process*	*8*	*6.96*	*0.04*	*ALDH1A1, CYP2J4, NCF1, CRABP2, PRKAB2, SGMS1, LPCAT2, OXSM*
GOTERM_BP_4	GO:0044255 ~ cellular lipid metabolic process	8	6.96	0.04	ALDH1A1, CYP2J4, NCF1, CRABP2, PRKAB2, SGMS1, LPCAT2, OXSM
*GOTERM_BP_3*	*GO:0006082* ~ *organic acid metabolic process*	*8*	*6.96*	*0.06*	*ALDH1A1, CYP2J4, SLC16A7, NCF1, CRABP2, PRKAB2, DDAH1, OXSM*
GOTERM_BP_4	GO:0043436 ~ oxoacid metabolic process	8	6.96	0.06	ALDH1A1, CYP2J4, SLC16A7, NCF1, CRABP2, PRKAB2, DDAH1, OXSM
GOTERM_BP_5	GO:0019752 ~ carboxylic acid metabolic process	8	6.96	0.05	ALDH1A1, CYP2J4, SLC16A7, NCF1, CRABP2, PRKAB2, DDAH1, OXSM
*GOTERM_BP_3*	*GO:0042180* ~ *cellular ketone metabolic process*	*8*	*6.96*	*0.07*	*ALDH1A1, CYP2J4, SLC16A7, NCF1, CRABP2, PRKAB2, DDAH1, OXSM*
*GOTERM_BP_3*	*GO:0006721* ~ *terpenoid metabolic process*	*3*	*2.61*	*0.02*	*ALDH1A1, CYP2J4, CRABP2*
GOTERM_BP_4	GO:0016101 ~ diterpenoid metabolic process	3	2.61	0.02	ALDH1A1, CYP2J4, CRABP2
GOTERM_BP_5	GO:0006721 ~ terpenoid metabolic process	3	2.61	0.02	ALDH1A1, CYP2J4, CRABP2
**GOTERM_BP_1**	**GO:0002376** **~** **immune system process**	**10**	**8.70**	**0.05**	**CD47, NCF1, C3, RT1-T18, KLRE1, CHRNA7, TRAF6, CD1D1, DDAH1, DPP4**
**GOTERM_BP_2**	**GO:0045321** **~** **leukocyte activation**	**5**	**4.35**	**0.06**	**KLRE1, CHRNA7, TRAF6, CD1D1, DPP4**
*GOTERM_BP_3*	*GO:0045321* ~ *leukocyte activation*	*5*	*4.35*	*0.05*	*KLRE1, CHRNA7, TRAF6, CD1D1, DPP4*
GOTERM_BP_4	GO:0046649 ~ lymphocyte activation	4	3.48	0.10	KLRE1, CHRNA7, CD1D1, DPP4
**GOTERM_BP_2**	**GO:0006955** **~** **immune response**	**7**	**6.09**	**0.06**	**CD47, NCF1, C3, RT1-T18, TRAF6, CD1D1, DDAH1**
**GOTERM_BP_2**	**GO:0002682** **~** **regulation of immune system process**	**7**	**6.09**	**0.04**	**CD47, C3, KLRE1, TRAF6, CD1D1, DDAH1, DPP4**
*GOTERM_BP_3*	*GO:0050776* ~ *regulation of immune response*	*6*	*5.22*	*0.02*	*C3, KLRE1, TRAF6, CD1D1, DDAH1, DPP4*
GOTERM_BP_4	GO:0050776 ~ regulation of immune response	6	5.22	0.01	C3, KLRE1, TRAF6, CD1D1, DDAH1, DPP4
GOTERM_BP_5	GO:0002253 ~ activation of immune response	4	3.48	0.02	C3, KLRE1, TRAF6, DDAH1
GOTERM_BP_4	GO:0002697 ~ regulation of immune effector process	5	4.35	0.01	C3, KLRE1, TRAF6, CD1D1, DPP4
GOTERM_BP_5	GO:0002697 ~ regulation of immune effector process	5	4.35	0.00	C3, KLRE1, TRAF6, CD1D1, DPP4
GOTERM_BP_5	GO:0002706 ~ regulation of lymphocyte mediated immunity	5	4.35	0.00	C3, KLRE1, TRAF6, CD1D1, DPP4
GOTERM_BP_5	GO:0002703 ~ regulation of leukocyte mediated immunity	5	4.35	0.00	C3, KLRE1, TRAF6, CD1D1, DPP4
GOTERM_BP_4	GO:0002819 ~ regulation of adaptive immune response	4	3.48	0.01	C3, TRAF6, CD1D1, DPP4
*GOTERM_BP_3*	*GO:0002682* ~ *regulation of immune system process*	*7*	*6.09*	*0.04*	*CD47, C3, KLRE1, TRAF6, CD1D1, DDAH1, DPP4*
GOTERM_BP_4	GO:0002684 ~ positive regulation of immune system process	6	5.22	0.02	CD47, C3, KLRE1, TRAF6, CD1D1, DDAH1
**GOTERM_BP_2**	**GO:0002252** **~** **immune effector process**	**4**	**3.48**	**0.05**	**CD47, NCF1, C3, DDAH1**
*GOTERM_BP_3*	*GO:0002252* ~ *immune effector process*	*4*	*3.48*	*0.05*	*CD47, NCF1, C3, DDAH1*
GOTERM_BP_4	GO:0006968 ~ cellular defense response	2	1.74	0.05	NCF1, DDAH1
**GOTERM_BP_1**	**GO:0022610** **~** **biological adhesion**	**7**	**6.09**	**0.09**	**CD47, SDC1, RET, CNTNAP1, COL5A3, BTBD9, CYR61**
**GOTERM_BP_2**	**GO:0007155** **~** **cell adhesion**	**7**	**6.09**	**0.09**	**CD47, SDC1, RET, CNTNAP1, COL5A3, BTBD9, CYR61**
**GOTERM_BP_1**	**GO:0048511** **~** **rhythmic process**	**7**	**6.09**	**0.00**	**ALDH1A1, NPAS2, JUND, PER2, TEF, CHRNA7, ARNTL**
**GOTERM_BP_2**	**GO:0007623 ~ circadian rhythm**	**4**	**3.48**	**0.01**	**NPAS2, JUND, PER2, ARNTL**

### Protein–protein interaction analysis

**Fig. 4:**
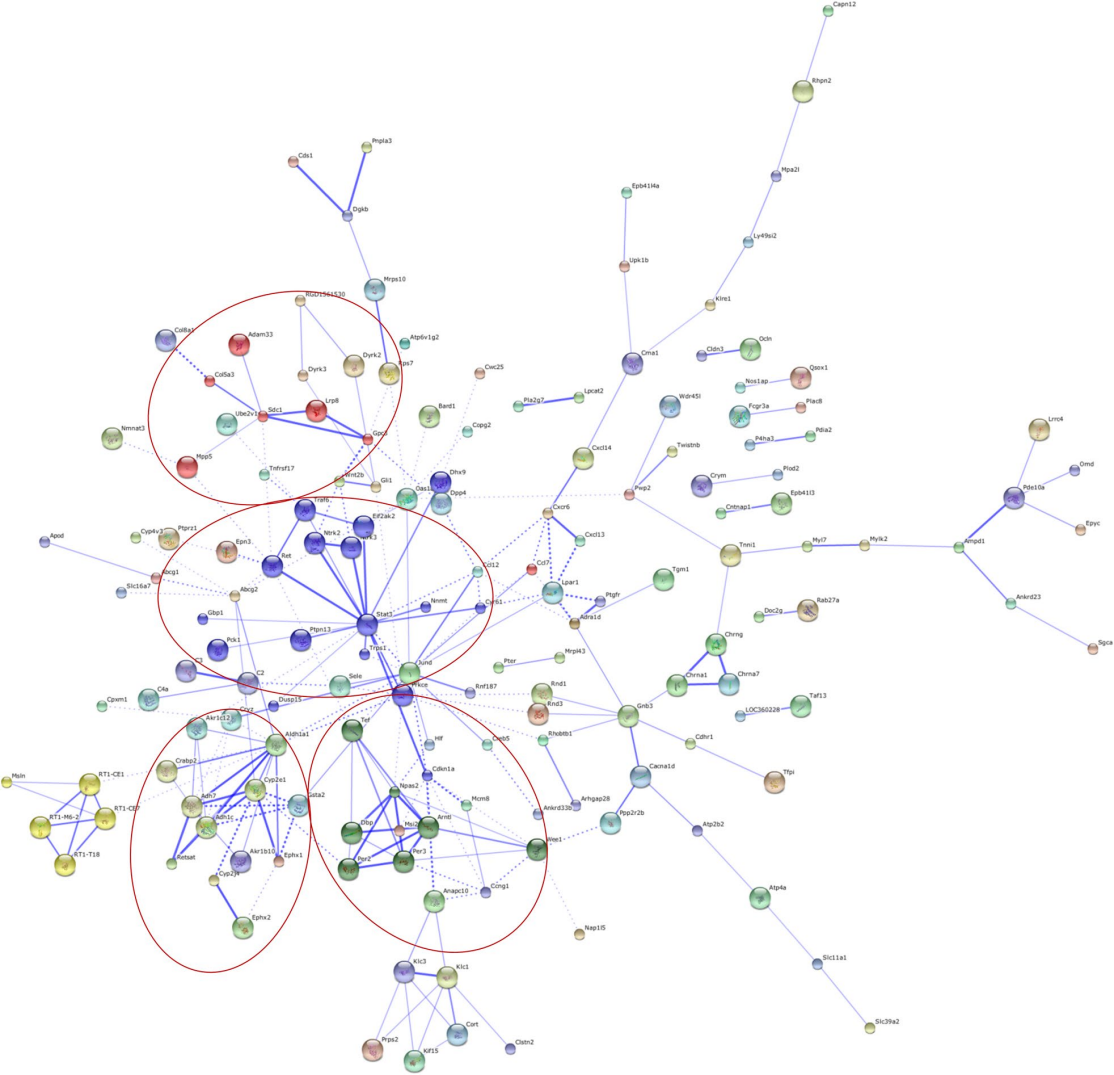
Protein–protein interaction networks. Predicted protein–protein interaction network of protein products of genes associated with non-obese T2DM. The *circle* stands for genes and the *line* indicates the interactions among genes. The *interior of the circle* represents the structure of proteins. The *thickness of the line* indicates the grade of evidence of the different interactions among proteins

To better understand the relationships between the functionally related gene groups analyzed by GO, we examined protein–protein interactions between protein products of all 507 genes showing significant difference in gene expression (Fig. 4). Here, Stat3 seems to have a major networking group affecting multiple top GO pathways in non-obese T2DM. Moreover, there are proteins interconnected with each other in smaller networking groups including networks of (1) Sdc1; (2) Cyp2e1 and (3) Tef proteins (Fig. 4) affecting the top GO pathways as well (Table 4).

## Discussion

In the present study, our aim was to investigate how cardiac gene expression pattern is influenced by non-obese T2DM. Here we show several characteristics of non-obese T2DM in 15 weeks old male GK rats including decreased body weight, fasting hyperglycemia, hypercholesterolemia, insulin resistance, and impaired glucose tolerance. Moreover, we show increased heart weight and heart weight/body weight ratio in GK rats indicating cardiac hypertrophy. We also demonstrate that non-obese T2DM influences cardiac gene expression pattern by altering transcript levels of several genes. We identified 507 genes which were differentially expressed in the myocardium of GK rats compared to Wistar controls.

The spontaneously diabetic GK rat is a well characterized and recognized model of non-obese type 2 diabetes mellitus. The abnormal glucose regulation in the GK rat develops in association both with impaired insulin secretion and with insulin resistance as reviewed by Östenson and Movassat [29, 32]. At the onset of T2DM, there is a compensatory attempt of the beta cells to release more insulin to defeat insulin resistance. Later this mechanism is insufficient to maintain blood glucose level within a physiological range and finally leads to the functional exhaustion of the surviving beta cells. These stages in the GK model could be also observed in our present study. Blood glucose levels were significantly increased in GK rats at weeks 7, 11 and 15 as compared to controls and there was a statistically significant difference in blood glucose levels in GK rats between the different time points. Blood glucose level in GK rats were significantly lower at week 11 as compared to week 7 or week 15 blood glucose values. Moreover, serum insulin level was significantly increased at week 11 in GK rats as compared to week 7 values. The significantly higher serum insulin level at week 11 could explain the lower blood glucose level at week 11 as compared to week 7 blood glucose values. Serum insulin levels and HOMA-IR were significantly increased at week 7 and 11 showing increasing insulin resistance and compensatory hyperinsulinemia. At week 15, there was no significant difference in serum insulin level between GK and control animals. However, pancreatic insulin content of GK rats was slightly decreased suggesting the functional exhaustion of pancreatic beta cells. Probably this is the reason why HOMA-IR failed to reach the level of statistical significance in GK rats compared to controls at this time point. Nevertheless, this is not a sign of spontaneous improvement of insulin resistance in GK rats in our present study. These results are in accordance with literature data showing that beta cell mass and insulin production continuously decreases from birth to adulthood in GK rats due to chronic islet inflammation, angiopathy, fibrosis and defective beta cell neogenesis [32].

Surprisingly, only a few studies were performed previously using the qPCR technique to investigate the gene expression changes playing a role in the development of left ventricular hypertrophy and structural remodeling [20, 35, 44], excitation–contraction coupling [45, 46], and lipotoxicity [10] in the hearts of GK rats. Thus, our study is the first to describe overall alterations in the cardiac transcriptome in male GK rats. In our present study, the significantly altered genes can be classified into different clusters (e.g. metabolism, stress response, signal transduction, regulation of transcription, receptors, ion channels, membrane and structural proteins, cell growth and differentiation, immune response, transport, hormones, etc.). Moreover, some other genes without any definite function in the myocardium were also changed in response to DM. The majority of these genes have not been related to non-obese T2DM yet, and therefore, characterization of the functional effects of these genes on the heart in non-obese T2DM is suggested in future mechanistic studies.

### Genes related to metabolic alterations in T2DM

In our present study, several genes related to metabolism were found to be affected in the hearts of GK rats as compared to controls. A group of these altered genes is involved in cellular ketone metabolic process according to GO and STRING protein–protein interaction analyses (e.g. down-regulation *of* cytochrome P450 2E1, (*Cyp2e1*); cytochrome P450 2J4, (*Cyp2j4*); and up-regulation of aldehyde dehydrogenase 1 family, member A1, (*Aldh1a1*); alcohol dehydrogenase 1, (*Adh1c*); aldo–keto reductase family 1, member B10, (*Akr1b10*); aldo–keto reductase family 1, member*, C12*, (*Akr1c12*); etc.) (Fig. 4; Table 4). It has been shown that 60 day old GK rats developed increased ketone body production [47], however, there is no literature data available about ketone body metabolism in the heart of GK rats. The ketone body acetone can be converted in vivo to glucose via acetol and pyruvate, and the initial conversion to acetol is catalyzed by *Cyp2e1* [48]. It has been shown that *Cyp2e1* knockout mice subjected to starvation to induce ketogenesis develop blood acetone levels much higher than those observed in wild-type mice [48]. In our present study, the down-regulation of *Cyp2e1* might be a possible cause of increased ketone body level in the myocardium, however, up-regulation of other genes involved in ketone metabolic process including *Aldh1a1*; *Adh1c*; *Akr1b10*; and *Akr1c12* may be an adaptive response in the myocardium to antagonize elevated ketone body levels. Moreover, *Akr1b10* has been shown to be inducible by hyperglycemia in peripheral blood mononuclear cells obtained from patients with diabetic nephropathy. It also could have potential downstream effect of reducing cellular retinoic acid level, which is a key molecule during organogenesis as well as the development of diabetic nephropathy [49]. In our present study, non-obese T2DM also influenced expression of genes related to epoxide metabolism (down-regulation of Epoxide hydrolase 2, cytoplasmic, (*Ephx2*); glutathione S-transferase alpha 2, (*Gsta2*) and up-regulation of Epoxide hydrolase 1, microsomal, (xenobiotic) (*Ephx1*) (Fig. 3). Epoxides are possible products of Cyp450 catalyzed oxidation of aromatic compounds which can lead to toxic, mutagenic or carcinogenic effects [50]. One possibility for their inactivation is the metabolism by epoxide hydrolases. In addition, glutathione-S-transferase catalyzed nucleophilic attack by glutathione may also lead to the inactivation of epoxides [50]. In our present study, *Cyp2e1*, *Cyp2j4*, *Ephx2* and *Gsta2* were down-regulated, showing decreased activity of epoxide metabolism. Moreover, glutathione S-transferase is a well-known enzyme catalyzing the conjugation of reduced glutathione on a wide variety of substrates including reactive oxygen and nitrogen species [51, 52]. Our results suggest that down-regulation of the anti-oxidative gene *Gsta2* may contribute to elevated myocardial oxidative/nitrative stress, a phenomenon that has been demonstrated in T2DM by several studies [53, 54]. Interestingly, we have found overexpression of glutathione S-transferase in the heart in streptozotocin-induced DM in neonatal rats [22], metabolic syndrome [21] and cholesterol diet-induced hyperlipidaemia [55] in our previous studies. Moreover, polymorphism of *Ephx2* has been shown to be a possible risk factor for developing insulin resistance and T2DM [56]. In addition, polymorphisms of another gene, the cellular retinoic acid binding protein 2 (*Crapb2,* down-regulated in our present study) has been reported to be a genetic marker of metabolic syndrome [57] and hypercholesterolemia [58].

### Genes related to diabetic cardiomyopathy

One of the major cardiovascular complications of DM is diabetic cardiomyopathy [59, 60], which is defined as left ventricular dysfunction with hypertrophy and fibrosis in the absence of hypertension, coronary artery disease and valvular or congenital heart disease [61]. The complex underlying molecular mechanisms of the above-mentioned functional and morphologic changes are not yet clear despite intensive investigations [59–61].

#### Structure elements

In our present study, we have shown altered expression of several genes playing a role in myocardial structure formation and potentially related to diabetic cardiomyopathy based on GO and STRING protein–protein interaction analyses. These altered genes include e.g. down-regulation of ADAM metallopeptidase domain 33 (*Adam33*); collagen, type V, alpha 3, (Col5a3); syndecan 1, (*Sdc1*); glypican 3 (*Gpc3*); troponin I type 1 (skeletal, slow) (*Tnni1*); myosin, light chain 7, regulatory (*Myl7*); myosin light chain kinase 2 (*Mylk2*) and up-regulation of collagen, type VIII, alpha 1, (*Col8a1*); etc. (Fig. 4; Table 4). Adam33 is a member of the ADAM protein family encoding a disintegrin and metalloprotease (ADAM) domain 33. It plays a role in cell–cell and cell–matrix interactions, including muscle development and neurogenesis and its polymorphism is associated with the development of T1DM [62]. Moreover, elevated type VIII collagen deposition in human diabetic nephropathy was demonstrated leading to the accumulation of extracellular matrix and periglomerular and interstitial fibrosis. Another ECM component, collagen V, is expressed as α1(V)2 α2(V) heterotrimers, which regulate collagen fibril geometry and strength in several tissues including the pancreas and skeletal muscle. Interestingly, skeletal muscle of Col5a3^−/−^ mice was defective in glucose uptake and mobilization of intracellular GLUT4 glucose transporter to the plasma membrane in response to insulin thereby leading to a glucose intolerant, insulin-resistant, and hyperglycemic phenotype [63]. Membrane proteoglycans Gpc3 and Sdc1 have not yet been demonstrated to play a role in the development of diabetic cardiomyopathy, however, increased expression of the heparan sulfate proteoglycans Gpc1 and Sdc4 has been shown to lead to diastolic dysfunction in streptozotocin-induced diabetic rats [64]. In addition, decreased expression of the genes playing a role in contractility including troponin I [65, 66], myosin light chain 7 [67] and myosin light chain kinase 2 [67] could lead to sarcomeric dysfunction and diabetic cardiomyopathy.

#### Receptors and ion channels

In our present study, several genes with receptor and/or ion channel function were found to be affected by non-obese T2DM and these genes might play a role in the development of diabetic cardiomyopathy. These genes include e.g. down-regulation *of* adrenoceptor alpha 1d (*Adra1d*); cholinergic receptor, nicotinic, gamma (muscle) (*Chrng*); cholinergic receptor, nicotinic, alpha 1 (*muscle*) (*Chrna1*); sarcolipin (*Sln*) and up-regulation *of* cholinergic receptor, nicotinic, alpha 7 (*neuronal*) (*Chrna7*); etc. Down-regulation of *Adra1d* receptor subtype has been previously shown in cardiac hypertrophy [68] and in STZ-induced DM by our research group [22]. Autonomic dysfunction is a serious complication of diabetes and can lead to cardiovascular abnormalities. It could be triggered by advanced glycation end products and reactive oxygen species mediated inactivation of neuronal nicotinic acetylcholine receptors, impairing synaptic transmission in sympathetic ganglia and resulting in autonomic failure [69]. Myocardial down-regulation of *Chrng* and *Chrna1* demonstrated in our present study might be another factor of cardiac autonomic dysfunction in diabetes. Moreover, it has been demonstrated that nicotinic cholinergic receptor alpha 7 (*Chrna7*) null mice showed decreased baroreflex-mediated tachycardia [70]. Sarcolipin (*Sln*) is a key regulator of sarcoplasmic reticulum Ca^2+^-ATPase (SERCA) and mediator of β-adrenergic responses [71]. It has been shown that *Sln*^−/−^ mice are susceptible to develop atrial arrhythmias and interstitial fibrosis due to altered expression of genes encoding collagen [71]. Ablation or mutation of *Sln* results in increased SERCA activity and Ca^2+^ load, causing abnormal intracellular Ca^2+^ handling and atrial remodeling with dysfunction [71, 72].

#### Signal transduction, regulation of transcription and biological processes

A major cluster of significantly altered cardiac genes in response to non-obese T2DM was associated with signal transduction, regulation of transcription and biological processes based on GO and STRING protein–protein interaction analyses (e.g. down-regulation of dipeptidylpeptidase 4 (*Dpp4*); signal transducer and activator of transcription 3 (acute-phase response factor) (*Stat3*); ret proto-oncogene transcript variant 1 (*Ret*); neurotrophic tyrosine kinase, receptor, type 2 (*Ntrk2*); neurotrophic tyrosine kinase, receptor, type 3 (*Ntrk3*) and up-regulation of Jun D proto-oncogene (*Jund*); etc.) (Fig. 4; Table 4). Dipeptidyl peptidase-4 is an integral membrane glycoprotein which cleaves N-terminal dipeptides from peptide molecules. The cleaved dipeptides are bioactive molecules regulating the cardiovascular system as well. Dpp4 inhibitors have been reported to be cardioprotective in most of the preclinical and clinical studies in T2DM [73]. In contrast, some cardiovascular outcome studies revealed increased hospitalization rates for heart failure among a subset of DPP4 inhibitor-treated diabetic subjects [74]. Recently a preclinical study has reported that diabetic mice treated with Dpp4 inhibitor exhibited modest cardiac hypertrophy, impairment of cardiac function, and dysregulated expression of genes and proteins controlling inflammation and cardiac fibrosis [75]. In our present study, *Dpp4* was down-regulated; therefore it does not seem to be a major and/or necessary regulator in the development of diabetic cardiomyopathy in non-obese T2DM. The transcription factor *Stat3* participates in a wide variety of physiological processes including proliferation, apoptosis, and cardiac survival especially during myocardial ischemia/reperfusion injury, however, its role is contradictory in these aforementioned processes [76]. Several studies have reported that cardiac *Stat3* and *phospho*-*Stat3* expression were reduced in diabetes, which can lead to cardiac dysfunction [76–78]. However, other studies reported that cardiac *Stat3* and *phospho*-*Stat3* expression were increased in diabetes leading to hypertrophy [79, 80]. Furthermore, it has been also reported that Stat3 deficient mice developed dilated cardiomyopathy [76] and patients with dilated cardiomyopathy had severely decreased myocardial Stat3 expression [81]. In the present study, Stat3 was down-regulated as assessed by microarray, however, qRT-PCR did not confirm these results in our hands. Expression of Stat3 might depend on the duration of DM and the stage of diabetic cardiomyopathy or heart failure. Moreover, there is no literature data available about *Stat3* expression in hearts of GK rats; therefore we could not compare our results to others. Surprisingly, down-regulation of *Tef* and *Ret* as well as up-regulation of *Jund* has not been shown previously to play a role in the development of diabetic cardiomyopathy. It has been reported that Ret-deficient mice exhibited a reduced volume of cardiac ganglia and cholinergic innervation of the ventricular conduction system [82]. JunD regulates genes involved in antioxidant defense and hydrogen peroxide (H_2_O_2_) production, as well as angiogenesis by controlling VEGF transcription [83]. Furthermore, an important function for JunD is to modulate insulin/insulin-like-growth factor 1 signaling and longevity [83]. Moreover, down-regulation of *Ntrk2* and *Ntrk3* genes might also play a role in the development of diabetic cardiomyopathy. Interestingly, it has been reported that mice with disrupted *Ntrk2* gene lacked a significant proportion of their intramyocardial blood vessels indicating that activation of the *Ntrk2* gene was crucial for normal vascularization of the developing heart [84]. In addition, mutations of *Ntrk3* gene have been shown in the development of human congenital heart diseases [85].

### Genes related to immune and antimicrobial response

A major cluster of significantly altered cardiac genes in response to non-obese T2DM in GK rats was associated with immune and antimicrobial response based on GO and STRING protein–protein interaction analyses (e.g. complement component 3 (C3); *complement component 4a* (*C4a*); chemokine (C–C motif) ligand 12 (Ccl12); chemokine (C-X-C motif) ligand 13 (*Cxcl13*); chemokine (C-X-C motif) ligand 14 (*Cxcl14*); chymase 1, mast cell (*Cma1*) and up-regulation of chemochine (*C*-*X*-*C* motif) receptor 6 (*Cxcr6*); killer cell lectin-like receptor, family E, member 1 (*Klre1*); etc.) which is in line with the well-known increased susceptibility to infections in DM [86, 87].

### Novel genes previously not related to diabetic alterations in the heart

Many of the genes showing altered expression in diabetic hearts in the present study have not yet been related to any diabetic alterations in the heart (e.g. down-regulation *of* low density lipoprotein receptor-related protein 8, apolipoprotein E receptor (*Lrp8*) and mesothelin (*Msln*) and up-regulation of kallikrein 1-related peptidase C3 (*Klk1c3*) and Epsin 3 (Epn3); etc.) (Table 4). Some other altered genes were not classified into specific functional clusters or indicated as yet uncharacterized, predicted genes and fragments (e.g. up-regulation of hydroxyacyl glutathione hydrolase-like and Similar to hepatic leukemia factor (*LOC690286*) or down-regulation of *uncharacterized LOC102546816* and similar to protein C17orf72; etc.) (Table 4), the relevance of which should not be ignored.

## Limitations

Our study is not without limitations. Insulin resistance was estimated in our study by determining HOMA-IR rather than the gold standard hyperinsulinemic euglycemic clamp technique. Nevertheless, the presence of insulin resistance in GK rats has been confirmed in several studies [88–91]. Although detailed morphological or histological analysis of GK hearts is lacking, our data including increased heart weight, heart weight to body weight ratio and coronary flow together with literature data suggest the development of LV hypertrophy in our model. Although our study does not specify which cell type (i.e. cardiomyocyte, fibroblast, smooth muscle cell, etc.) may be responsible for the observed alterations of cardiac gene expression due to DM, the contribution of cardiomyocytes is likely the most significant [92, 93]. In addition, it is unclear whether significantly altered gene expression changes at the mRNA level are further translated to changes in protein levels and if gene expression changes are causes or consequences of the development of diabetic cardiomyopathy, therefore, additional in-depth mechanistic studies should be carried out.

## Conclusions

In summary, we have found that 15 week old male GK rats develop non-obese T2DM and we have demonstrated for the first time that non-obese T2DM is associated with a profound modification of the cardiac transcriptome. Some of the genes showing altered expression in the hearts of GK rats have been implicated in non-obese T2DM previously by other techniques. Some of the genes showing altered expression in our present study in non-obese T2DM have been reported to be associated with cardiac alterations in obese T2DM or T1DM models. Many of the genes showing significant expressional alterations in GK rat hearts in the present study have not been associated with non-obese T2DM previously. We conclude that non-obese T2DM alters the gene expression pattern of the myocardium. These altered genes may be involved in the development of cardiac pathologies in the state of non-obese type 2 diabetes mellitus. Based on our exploratory results, future studies should be carried out to investigate the precise role of specific genes in the development of cardiac consequences of non-obese T2DM to obtain deeper mechanistic insight.

## Additional files

10.1186/s12933-016-0424-3 Raw data of the DNA microarray.
10.1186/s12933-016-0424-3 Additional Tables.

## Abbreviations

Adra1d: adrenoceptor alpha 1d
Adam33: a disintegrin and metalloprotease domain 33
Adh1c: alcohol dehydrogenase 1
Akr1b10: aldo-keto reductase family 1, member B10
Akr1c12: aldo-keto reductase family 1, member, C12
Aldh1a1: aldehyde dehydrogenase 1 family, member A1
ANOVA: analysis of variance
AUC: area under the curve
C3: complement component 3
C4a: complement component 4a
Ccl12: chemokine (C–C motif) ligand 12
cDNA: complementary deoxyribonucleic acid
Chrna1: cholinergic receptor, nicotinic, alpha 1 (muscle)
Chrna7: cholinergic receptor, nicotinic, alpha 7 (neuronal)
Chrng: cholinergic receptor, nicotinic, gamma (muscle)
Cma1: chymase 1, mast cell
Col5a3: collagen, type V, alpha 3
Col8a1: collagen, type VIII, alpha 1
Crapb2: cellular retinoic acid binding protein 2
Cxcl13: chemokine (C-X-C motif) ligand 13
Cxcr6: chemochine (C-X-C motif) receptor 6
Cyp2e1: cytochrome P450 2E1
Cyp2j4: cytochrome P450 2J4
DAVID: Database for Annotation, Visualization and Integrated Discovery
DCM: diabetic cardiomyopathy
DNA: deoxyribonucleic acid
Dpp4: dipeptidylpeptidase 4
ELISA: enzyme linked immunosorbent assay
Ephx1: epoxide hydrolase 1, microsomal, (xenobiotic)
Ephx2: epoxide hydrolase 2, cytoplasmic
Epn3: epsin 3
GAPDH: glyceraldehyde-3-phosphate dehydrogenase
GK rat: Goto-Kakizaki rat
GLUT4: glucose transporter 4
GO: gene ontology
Gpc3: glypican 3
Gsta2: glutathione S-transferase alpha 2
HOMA-IR: homeostasis model assessment-estimated insulin resistance
HPRT: hypoxanthine phosphoribosyl transferase
Jund: Jun D proto-oncogene
Klk1c3: kallikrein 1-related peptidase C3
Klre1: killer cell lectin-like receptor, family E, member 1
Lrp8: low density lipoprotein receptor-related protein 8, apolipoprotein E receptor
LV: left ventricular
mRNA: messenger ribonucleic acid
Msln: mesothelin
Myl7: myosin, light chain 7, regulatory
Mylk2: myosin light chain kinase 2
Ntrk: neurotrophic tyrosine kinase, receptor
OGTT: oral glucose tolerance test
qRT-PCR: quantitative real-time polymerase chain reaction
Ret: ret proto-oncogene transcript variant 1
RNA: ribonucleic acid
RPS18: ribosomal protein S18
Sdc1: syndecan 1
SEM: standard error of mean
SERCA: sarcoplasmic reticulum Ca^2+^-ATPase
Sln: sarcolipin
Stat3: signal transducer and activator of transcription 3
STZ: streptozotocin
T1DM: type 1 diabetes mellitus
T2DM: type 2 diabetes mellitus
Tnni1: troponin I type 1 (skeletal, slow)
ZDF rat: Zucker Diabetic Fatty rat
