# Supplementary material for: Transcriptomic alterations in the heart of non-obese type 2 diabetic Goto-Kakizaki rats
Source: Cardiovasc Diabetol. 2016 Aug 5;15:110. doi: 10.1186/s12933-016-0424-3 (PMC4975916; doi:10.1186/s12933-016-0424-3)
Supplement: Supplementary file 2 — 10.1186/s12933-016-0424-3 Additional Tables. [file 12933_2016_424_MOESM2_ESM.docx]

**Table S2** Up-regulated genes (>2.0-fold up-regulation)

**Table S2 (continued)** Up-regulated genes (>2.0-fold up-regulation)

**Table S2 (continued)** Up-regulated genes (>2.0-fold up-regulation)

**Table S3** Down-regulated genes (>2.0-fold down-regulation)

**Table S3** **(continued)** Down-regulated genes (>2.0-fold down-regulation)

| **Gene function** | **Description (Gene symbol)** | **Acc. No.:** | **AVE log2** | **SD (LOG)** | **P value (Corr)** | **Fold change** | **SD** |
| --- | --- | --- | --- | --- | --- | --- | --- |
| **Others** | Similar to Hypothetical protein KIAA1340 (LOC300789) | XM_236350 | -1.01 | 0.59 | 0.0216 | -2.01 | 1.04 |
|  | Leucine rich repeat containing 46 (Lrrc46) | NM_001004201 | -1.02 | 0.31 | 0.0006 | -2.03 | 0.54 |
|  | Crystallin, gamma D (Crygd) | NM_033095 | -1.02 | 0.25 | 0.0006 | -2.03 | 0.42 |
|  | Musashi RNA-binding protein 2 (Msi2) | XM_006247074 | -1.02 | 0.19 | 0.0011 | -2.03 | 0.31 |
|  | Tetranectin-like (LOC100912012) | XM_003750609 | -1.03 | 0.63 | 0.0072 | -2.04 | 1.13 |
|  | HECT, UBA and WWE domain containing 1 (Source:MGI Symbol;Acc:MGI:1926884) | XR_349618 | -1.02 | 0.32 | 0.0001 | -2.03 | 0.57 |
|  | similar to Discs large homolog 5 (Placenta and prostate DLG) (Discs large protein P-dlg) (LOC685792) | XM_217497 | -1.03 | 0.33 | 0.0007 | -2.04 | 0.58 |
|  | Major Facilitator Superfamily protein | BG666908 | -1.04 | 0.59 | 0.0144 | -2.05 | 1.06 |
|  | similar to RIKEN cDNA 1700001E04 (MGC116197) | NM_001025755 | -1.04 | 0.25 | 0.0002 | -2.06 | 0.46 |
|  | pantothenate kinase 2 (Pank2) | XM_006235020 | -1.04 | 0.48 | 0.0060 | -2.06 | 0.94 |
|  | TL0ADA46YP04 mRNA sequence | FQ229724 | -1.05 | 0.22 | 0.0001 | -2.07 | 0.36 |
|  | uncharacterized LOC100909904 (LOC100909904) | XR_146747 | -1.06 | 0.56 | 0.0185 | -2.08 | 1.05 |
|  | uncharacterized LOC100909776 (LOC100909776) | XM_006225427 | -1.07 | 0.50 | 0.0048 | -2.10 | 0.88 |
|  | paired immunoglobulin-like type 2 receptor alpha-like (LOC100910669) | XM_003751149 | -1.09 | 0.37 | 0.0001 | -2.13 | 0.61 |
|  | mast cell protease 8-like (LOC100912724) | XM_003752804 | -1.10 | 0.78 | 0.0254 | -2.14 | 1.91 |
|  | similar to RIKEN cDNA 4933431D05 (LOC363424), mRNA (XM_343747) | XM_343747 | -1.11 | 0.49 | 0.0012 | -2.15 | 1.00 |
|  | BP489994 insulinoma RINm5F cDNA clone RI07274 3' | BP489994 | -1.13 | 0.28 | 0.0008 | -2.19 | 0.55 |
|  | cdig1U mRNA for hypothetical protein | AB086233 | -1.13 | 0.21 | 0.0001 | -2.19 | 0.39 |
|  | mitochondrial ribosomal protein L43 (Mrpl43) | NM_001107598 | -1.13 | 0.29 | 0.0001 | -2.19 | 0.53 |
|  | similar to Protein C17orf72 (RGD1307288) | XM_001081575 | -1.14 | 0.58 | 0.0042 | -2.20 | 1.08 |
|  | similar to RIKEN cDNA 3110035E14 (RGD1561849) | NM_001109260 | -1.14 | 0.48 | 0.0008 | -2.21 | 1.02 |
|  | Protein Enox1; RCG36864 (Source:UniProtKB/TrEMBL;Acc:D3ZYM3) | XM_006222034 | -1.14 | 0.71 | 0.0105 | -2.21 | 1.46 |
|  | angiotensin-converting enzyme-like (LOC102556346) | XM_006247643 | -1.15 | 0.31 | 0.0002 | -2.22 | 0.57 |
|  | Cytochrome P450 family protein |  | -1.17 | 0.67 | 0.0032 | -2.24 | 1.18 |
|  | Uncharacterized protein (Source:UniProtKB/TrEMBL;Acc:F1LTE7) (ENSRNOT00000059208) |  | -1.18 | 0.94 | 0.0481 | -2.26 | 2.06 |
|  | Pva1 protein |  | -1.18 | 0.45 | 0.0003 | -2.27 | 0.81 |
|  | elongation of very long chain fatty acids protein 6-like (LOC102549542) | XM_006233301 | -1.20 | 0.77 | 0.0072 | -2.29 | 1.37 |
|  | centrosomal protein 19 (Cep19) | NM_138865 | -1.20 | 0.24 | 0.0000 | -2.30 | 0.45 |
|  | hypothetical protein LOC679342 (LOC679342) | XM_006222359 | -1.21 | 0.22 | 0.0001 | -2.31 | 0.43 |
|  | PWP2 periodic tryptophan protein homolog (yeast) (Pwp2) | XM_006256257 | -1.23 | 0.37 | 0.0001 | -2.34 | 0.64 |
|  | THAP domain containing 6 (Thap6) | NM_001107209 | -1.25 | 0.46 | 0.0001 | -2.38 | 0.87 |
|  | uncharacterized LOC100910374 (LOC100910374) | XR_146227 | -1.25 | 0.23 | 0.0000 | -2.38 | 0.49 |
|  | similar to Discs large homolog 5 (Placenta and prostate DLG) (Discs large protein P-dlg) (LOC691666) | XR_358222 | -1.26 | 0.44 | 0.0002 | -2.40 | 0.82 |
|  | Coiled-coil domain containing 68 (Ccdc68) | NM_001077667 | -1.29 | 0.92 | 0.0196 | -2.44 | 1.80 |
|  | rho GTPase-activating protein 20-like (LOC102556298) | XM_006244397 | -1.29 | 0.45 | 0.0003 | -2.44 | 0.93 |
|  | Protein LOC100360856 (Source:UniProtKB/TrEMBL;Acc:M0R7H3) (ENSRNOT00000071982) | XM_006227129 | -1.29 | 0.31 | 0.0001 | -2.45 | 0.63 |
|  | uncharacterized LOC100909739 (LOC100909739) | XR_146366 | -1.29 | 0.42 | 0.0009 | -2.45 | 0.92 |
|  | Q4S355_TETNG (Q4S355) Chromosome 4 SCAF14752, whole genome shotgun sequence |  | -1.30 | 0.12 | 0.0002 | -2.47 | 0.26 |
|  | uncharacterized LOC102546816 (LOC102546816) | XR_356810 | -1.32 | 0.44 | 0.0005 | -2.50 | 0.96 |
|  | uncharacterized LOC102552673 (LOC102552673) | XR_347380 | -1.32 | 0.96 | 0.0275 | -2.50 | 2.09 |
|  | uncharacterized LOC102551154 (LOC102551154) | XR_361579 | -1.33 | 0.42 | 0.0003 | -2.51 | 0.83 |
|  | similar to RIKEN cDNA 1700001E04 (LOC363379), mRNA (XM_343714) | XM_343714 | -1.33 | 0.38 | 0.0012 | -2.52 | 0.86 |
|  | Q7TMA9_RAT (Q7TMA9) Ac1262 (Ac1-114) (Ac2-069) (Ab2-196) (Ab1-341) (Ba2-693) (Aa1249) | XM_343740 | -1.35 | 0.35 | 0.0004 | -2.55 | 0.80 |
|  | keratin 8-like (LOC100362689) | XR_146223 | -1.37 | 0.37 | 0.0003 | -2.58 | 0.85 |
|  | secretogranin V (7B2 protein) (Scg5) | NM_013175 | -1.38 | 0.49 | 0.0012 | -2.59 | 1.05 |
|  | disks large homolog 5-like (LOC100912852) | XM_006227129 | -1.38 | 0.28 | 0.0004 | -2.61 | 0.64 |
|  | 1304303B clathrin LCa. {Bos taurus} (exp=-1; wgp=0; cg=0) |  | -1.38 | 0.34 | 0.0000 | -2.61 | 0.76 |
|  | placenta-specific 8 (Plac8) | NM_001108353 | -1.41 | 0.47 | 0.0001 | -2.65 | 0.98 |
|  | uncharacterized LOC102551154 (LOC102551154) | XR_361579 | -1.41 | 0.39 | 0.0001 | -2.66 | 0.84 |
|  | double C2-like domains, gamma (Doc2g) | NM_001011937 | -1.41 | 0.38 | 0.0001 | -2.66 | 0.77 |
|  | glutamine rich 2 (Qrich2) | XM_006220926 | -1.42 | 0.61 | 0.0007 | -2.67 | 1.20 |
|  | similar to RIKEN cDNA 1700001E04 (MGC116197) | NM_001025755 | -1.44 | 0.32 | 0.0001 | -2.71 | 0.79 |
|  | Protein LOC100360856 | XM_006227084 | -1.46 | 0.29 | 0.0000 | -2.74 | 0.72 |
|  | CD99 antigen-like protein 2-like (LOC102557427) | XM_006227145 | -1.46 | 0.41 | 0.0002 | -2.75 | 1.01 |
|  | BRCA1 associated RING domain 1 (Bard1) | NM_022622 | -1.49 | 0.19 | 0.0001 | -2.81 | 0.44 |
|  | EF-hand domain family, member D1 (Efhd1) | NM_001109310 | -1.49 | 0.42 | 0.0000 | -2.82 | 1.09 |
|  | CTF8, chromosome transmission fidelity factor 8 homolog (S. cerevisiae) (Chtf8) | NM_001194951 | -1.49 | 0.77 | 0.0033 | -2.82 | 1.61 |
|  | Uncharacterized protein (Source:UniProtKB/TrEMBL;Acc:D3ZK18) (ENSRNOT00000061865) |  | -1.53 | 0.29 | 0.0000 | -2.90 | 0.75 |
|  | WAP four-disulfide core domain 1 (Wfdc1) | NM_133581 | -1.54 | 0.60 | 0.0034 | -2.90 | 1.38 |
|  | hypothetical gene supported by AF152002 (LOC290595) | NM_001106063 | -1.54 | 0.97 | 0.0206 | -2.91 | 3.23 |
|  | Rat VL30 element | M91234 | -1.55 | 0.62 | 0.0002 | -2.93 | 1.70 |
|  | Protein LOC690251 | XM_001073854 | -1.58 | 0.33 | 0.0000 | -2.98 | 0.83 |
|  | LOC362451 (RGD1306750) | NM_001108649 | -1.58 | 0.51 | 0.0006 | -2.99 | 1.30 |
|  | Protein Arhgef5 (Source:UniProtKB/TrEMBL;Acc:E9PT59) | XM_006224892 | -1.62 | 0.54 | 0.0002 | -3.07 | 1.33 |
|  | hypothetical protein LOC100302372 (LOC100302372) | NM_001162897 | -1.63 | 0.57 | 0.0004 | -3.10 | 1.69 |
|  | (F344/Crj)rearranged mRNA for T-cell receptor gamma chain (1483bp) | Z27087 | -1.65 | 0.71 | 0.0045 | -3.13 | 1.94 |
|  | similar to RIKEN cDNA 1700001E04 (LOC367428), mRNA (XM_346135) | XM_346135 | -1.65 | 0.25 | 0.0000 | -3.14 | 0.68 |
|  | BTB (POZ) domain containing 9 (Btbd9) | XM_006256185 | -1.66 | 0.30 | 0.0000 | -3.15 | 0.73 |
|  | Q99NG8_RAT (Q99NG8) T:G mismatch thymine glycosylase |  | -1.67 | 0.44 | 0.0001 | -3.17 | 1.24 |
|  | hypothetical protein LOC689316 (LOC689316) | XR_086061 | -1.68 | 0.39 | 0.0000 | -3.21 | 1.02 |
|  | uroplakin 3B-like (Upk3bl) | NM_001109020 | -1.69 | 0.37 | 0.0013 | -3.22 | 1.04 |
|  | NEUU_MOUSE (Q9QXK8) Neuromedin U-23 precursor (NmU-23) |  | -1.71 | 0.43 | 0.0000 | -3.28 | 1.10 |
|  | ripply transcriptional repressor 2 (Ripply2) | XM_001064780 | -1.73 | 0.44 | 0.0000 | -3.31 | 1.22 |
|  | uncharacterized LOC100912446 (LOC100912446) | FQ221838 | -1.73 | 0.64 | 0.0005 | -3.33 | 1.76 |
|  | similar to TP53-regulating kinase (p53-related protein kinase) (Nori-2) (LOC685619) | XM_002729250 | -1.75 | 0.32 | 0.0000 | -3.37 | 0.84 |
|  | erythrocyte membrane protein band 4.1-like 3 (Epb41l3) | NM_053927 | -1.77 | 0.90 | 0.0057 | -3.41 | 2.69 |
|  | uncharacterized LOC102556259 (LOC102556259) | XR_355327 | -1.77 | 0.38 | 0.0001 | -3.42 | 1.22 |
|  | EF-hand domain family, member D1 (Efhd1) | NM_001109310 | -1.81 | 0.46 | 0.0000 | -3.50 | 1.45 |
|  | zinc finger and BTB domain containing 20 (Zbtb20) | XM_006248302 | -1.87 | 0.48 | 0.0000 | -3.67 | 1.69 |
|  | suppressor of glucose, autophagy associated 1 (Soga1) | XM_001067659 | -1.89 | 0.66 | 0.0001 | -3.72 | 2.23 |
|  | Protein RGD1562667 | XM_221091 | -1.91 | 0.45 | 0.0000 | -3.75 | 1.49 |
|  | Uncharacterized protein (Source:UniProtKB/TrEMBL;Acc:F1LSJ2) (ENSRNOT00000035259) | XM_001061015 | -1.93 | 0.61 | 0.0001 | -3.80 | 2.07 |
|  | uncharacterized LOC102546664 (LOC102546664) | XR_342060 | -1.95 | 0.18 | 0.0000 | -3.87 | 0.58 |

**Table S3** **(continued)** Down-regulated genes (>2.0-fold down-regulation)
